# Pannexin 1 and Pannexin 3 Channels Regulate Skeletal Muscle Myoblast Proliferation and Differentiation*

**DOI:** 10.1074/jbc.M114.572131

**Published:** 2014-09-19

**Authors:** Stéphanie Langlois, Xiao Xiang, Kelsey Young, Bryce J. Cowan, Silvia Penuela, Kyle N. Cowan

**Affiliations:** From the ‡ Department of Surgery, Division of Paediatric Surgery, University of Ottawa, Children's Hospital of Eastern Ontario, Ottawa, Ontario K1H 8L1, Canada,; § Apoptosis Research Center, Children's Hospital of Eastern Ontario, Ottawa, Ontario K1H 8L1, Canada,; ¶ Department of Cellular and Molecular Medicine, University of Ottawa, Ottawa, Ontario K1H 8L1, Canada,; ‖ Department of Dermatology and Skin Science, University of British Columbia, Vancouver, British Columbia V5Z 4E8, Canada, and; ** Department of Anatomy and Cell Biology, University of Western Ontario, London, Ontario N6A 5C1, Canada

**Keywords:** Differentiation, Myogenesis, Pannexin, Proliferation, Skeletal Muscle, Myoblasts

## Abstract

Pannexins constitute a family of three glycoproteins (Panx1, -2, and -3) forming single membrane channels. Recent work demonstrated that Panx1 is expressed in skeletal muscle and involved in the potentiation of contraction. However, Panxs functions in skeletal muscle cell differentiation, and proliferation had yet to be assessed. We show here that Panx1 and Panx3, but not Panx2, are present in human and rodent skeletal muscle, and their various species are differentially expressed in fetal *versus* adult human skeletal muscle tissue. Panx1 levels were very low in undifferentiated human primary skeletal muscle cells and myoblasts (HSMM) but increased drastically during differentiation and became the main Panx expressed in differentiated cells. Using HSMM, we found that Panx1 expression promotes this process, whereas it was impaired in the presence of probenecid or carbenoxolone. As for Panx3, its lower molecular weight species were prominent in adult skeletal muscle but very low in the fetal tissue and in undifferentiated skeletal muscle cells and myoblasts. Its overexpression (∼43-kDa species) induced HSMM differentiation and also inhibited their proliferation. On the other hand, a ∼70-kDa immunoreactive species of Panx3, likely glycosylated, sialylated, and phosphorylated, was highly expressed in proliferative myoblasts but strikingly down-regulated during their differentiation. Reduction of its endogenous expression using two Panx3 shRNAs significantly inhibited HSMM proliferation without triggering their differentiation. In summary, our results demonstrate that Panx1 and Panx3 are co-expressed in human skeletal muscle myoblasts and play a pivotal role in dictating the proliferation and differentiation status of these cells.

## Teaser

**Background:** Pannexins functions in skeletal myogenesis are unknown.

**Results:** Panx1 and Panx3 species are co-expressed in skeletal muscle. Their levels are modulated during myoblast differentiation regulating either myoblast proliferation and/or differentiation status.

**Conclusion:** Panx1 and Panx3 channels are novel regulators of skeletal muscle myoblast differentiation and proliferation.

**Significance:** Skeletal muscle development and health depend on functional pannexin channels.

## Introduction

The pannexin (Panx)^2^ family of channel proteins consists of three members in the mammalian genome: Panx1, Panx2, and Panx3 (1). Panx1 is ubiquitously expressed in many organs and tissues, whereas Panx2 seems to be primarily found in the brain. On the other hand, Panx3 is most abundant in skin, bone, and cartilage (2). Both Panx1 and Panx3 are heavily glycosylated, generating distinct species in rat and mice (3–5). Their glycosylation has been found to play a role in their proper trafficking and cellular localization as well as in their intermixing, which can potentially regulate their channel function (3–5). Panx3, with an expected molecular mass of ∼43–44 kDa, exhibits an immunoreactive species at ∼70 kDa in several tissues and cell types (3, 6–8). Panxs have been shown to form single membrane channels involved in ATP release into the extracellular space as well as functioning as Ca^2+^ channels in the ER (for review see Ref. 1).

There is now increasing evidence that Panx1 channels are involved in many cellular and physiological functions such as in long range Ca^2+^ wave propagation (9), vasodilatation (10), inflammatory responses (11, 12), ischemic death of neurons (13), death of enteric neurons during colitis (14), epilepsy (15), apoptosis (16, 17), stabilization of synaptic plasticity and learning (18), keratinocyte differentiation (6), and carcinogenesis (1, 7, 19, 20). As opposed to Panx1, much less is known about the cellular and physiological functions of Panx3. We have recently reported that Panx3 levels are altered in human keratinocyte tumors, suggesting a role in carcinogenesis (7). Studies have also reported a role for Panx3 in regulating chondrocytes (21) and osteoprogenitor cell proliferation (22) as well as in promoting osteoblast (23) and chondrocyte (21) differentiation.

It is only recently that pannexins have been studied in skeletal muscle. In addition to its expression at the mRNA level in human skeletal muscle (24), Panx1 protein has been shown to be localized at the sarcolemma (25) and the T-tubules of adult rodent skeletal muscle fibers (25, 26). During the contraction of skeletal muscle, ATP is released from the muscle fibers. In skeletal muscle myotubes in culture, ATP release during tetanic stimulation was abolished by the inhibition of Panx1 channels (27). It was later demonstrated that the ATP release induced by repetitive electrical stimulation was inhibited by Panx channel blockers (25, 26) and did not occur in the soleus muscle of Panx1 knock-out mice (26). Although Panx1 may be involved in the potentiation of contraction (26, 27), possible functions for Panxs in skeletal muscle cell differentiation and proliferation have yet to be assessed.

Here we show that in addition to Panx1, Panx3 is also expressed in skeletal muscle cells and tissue and that the protein levels of both Panxs are regulated during skeletal muscle differentiation. Panx1 levels are dramatically induced during human primary skeletal muscle myoblast differentiation and indeed promote this process. Accordingly, the Panx channel blockers probenecid and carbenoxolone (CBX) interfered with skeletal myogenic differentiation. Panx3 (∼43 kDa) was mainly expressed in differentiated human skeletal muscle tissue, and although its expression accelerated the differentiation of skeletal muscle myoblasts, it also inhibited their proliferation. On the other hand, the ∼70-kDa immunoreactive species of Panx3, which is likely glycosylated, sialylated, and phosphorylated, was highly expressed in fetal skeletal muscle tissue and undifferentiated skeletal muscle cells and was drastically down-regulated during differentiation. Successful knockdown of this species using Panx3 shRNAs further suggests that this glycoprotein corresponds to a form of Panx3. Reduction of its endogenous expression significantly inhibited myoblast proliferation without triggering differentiation. Collectively, our results reveal that both Panx1 and Panx3 are expressed in skeletal muscle myoblasts and that their levels are differentially modulated during the differentiation process regulating either myoblast proliferation and/or differentiation status.

## EXPERIMENTAL PROCEDURES

### 

#### 

##### Primary Cells, Cell Lines, and Culture Conditions

Primary human skeletal muscle myoblasts (HSMM; from post gestational tissue, usually from the quadriceps or psoas tissue) and primary human skeletal muscle cells (SkMC; isolated from the upper arm or leg skeletal muscle tissue) were propagated as specified by the supplier (Lonza, Walkersville, MD) and maintained in Clonetics SkGM-2 and SkGM Bullet kit (Lonza), respectively. HEK293T cells were from the ATCC (Manassas, VA) and maintained in DMEM + 10% FCS.

##### Plasmids and Transfections

Untagged Panx1, Panx2, and Panx3 expression constructs were previously described (3, 4). Cells were transfected using Lipofectamine 2000 (Invitrogen).

##### Mouse, Rat, and Human Tissue Samples for Western Blotting

Quadriceps from adult (7 months old) BL6 mice and Sprague-Dawley rats were snap-frozen for Western blot analysis. Samples were homogenized in 1% SDS (in PBS) containing proteases and phosphatases inhibitors using the Omni Bead Ruptor with 2.38-mm stainless steel beads. Normal fetal and adult human skeletal muscle whole lysates were obtained from Novus Biologicals (Oakville, ON, Canada).

##### Immunolabeling of Panx1 and Panx3 in Human Skeletal Muscle Sections

Human striated skeletal muscle (piloerector muscle) in skin samples were obtained after informed consent from patients attending the Vancouver General Hospital Skin Care Center, University of British Columbia, Vancouver, British Columbia, Canada, and performed in accordance with the ethical principles set for in the Declaration of Helsinki and at the University of British Columbia. Samples were fixed in 10% neutral buffered formalin and embedded in paraffin.

Sections (5 μm thick) were then deparaffinized in xylene and rehydrated in graded alcohols. For Panx1, antigen retrieval was done using Vector Antigen Unmasking Solution (Vector Laboratories, Burlingame, CA) according to the manufacturer's instructions. Antigen retrieval using sodium citrate buffer was used for Panx3 (3, 7). Tissue sections were labeled with anti-human Panx1 (1:50) or anti-Panx3 (CT-379, 1:50) (3, 7). Appropriate secondary antibodies conjugated to Texas-Red (Jackson ImmunoResearch, West Grove, PA, 1:100) were applied. A peptide pre-adsorption assay was done to verify antibody specificity as previously described (3, 7). Hoechst 33342 (Molecular Probes, Eugene, OR) was used as a nuclear stain, and labeling was visualized with a Zeiss LSM 510 META inverted confocal microscope.

##### Western Blotting

HSMM, SkMC, and HEK293T cell lysates were obtained as previously described (7). After separation by SDS-PAGE, proteins were transferred to PVDF membranes and immunoblotted with anti-Panx1 (3), anti-Panx2 (28), anti-Panx3 (3), anti-desmin (Cell Signaling, Whitby, ON, Canada), anti-MHC (R&D Systems, Minneapolis, MN), and anti-PCNA (Dako, Burlington, ON, Canada). Alexa 680 (Invitrogen) or infrared fluorescent-labeled secondary antibodies (1:5000) (IRDye 800 (Rockland Immunochemicals, Gilbertsville, PA)) were used, and immunoblots were quantified using the Odyssey infrared-imaging system (Licor). The membranes were reprobed for tubulin for normalization of protein loading. Protein standards are depicted in kDa. The relative intensities of the bands represent the average ± S.D. of at least three independent Western blots. Lysates from HEK293T cells overexpressing either Panx1, Panx2, or Panx3 have been used as positive controls.

##### Deglycosylation Assay

Deglycosylation assays were performed as described previously (3, 7). Briefly, equal amounts of protein lysates from human skeletal muscle tissue lysates as well as HSMM or HEK293T transfected with Panx1 or Panx3 were subjected to deglycosylation treatment with or without the enzyme mix following the manufacturer's instructions (New England Biolabs). Samples (with or without enzymes) were incubated for 4 h at 37 °C. In another set of experiments, samples were processed for deglycosylation using individual enzymes following the manufacturer's instructions (Prozyme/Glyco). Briefly, HSMM lysates were heated in the provided denaturation buffer solution for 5 min at 100 °C. Enzymes (1 μl) were added and incubated at 37 °C for 3 h. The control lysates (without enzymes) were also incubated at 37 °C for the same period of time. After incubation at 37 °C, samples were boiled and separated on 10% SDS-PAGE. Gels were transferred onto PVDF membranes and processed for immunoblotting with anti-Panx1 or anti-Panx3 antibody to detect a shift in the banding pattern of any Panx species upon deglycosylation.

##### Chemical Cross-linking

The following protocol was slightly modified from the previously described method used to cross-link Panx1 monomers within the oligomers (5). Wild-type HEK293T or HEK293T cells transfected with Panx3 were washed 3 times with cold PBS, scraped, and centrifuged at 11,500 × *g* for 10 min at 4 °C. The cell pellet was resuspended in cold PBS and sonicated on ice (3 pulses, 30 s each). Triton X-100 was then added to a final concentration of 1%. Samples were incubated on ice for 30 min and centrifuged at 11,500 × *g* for 10 min at 4 °C. The supernatant was kept on ice and used for the cross-linking assay.

Cross-linking using dithiobis(succinimidylpropionate) (DSP) was performed following the manufacturer's instructions (Thermo Scientific). A DSP solution was prepare just before use (12.5 mg/ml) and added to a final concentration of 300 μg/ml, mixed, and incubated at room temperature for 30 min. The reaction was stopped by the addition of 1 m Tris, pH 7.5. The samples were then mixed and incubated for 15 min. To reverse the cross-linking, samples were boiled in the presence of 5% β-mercaptoethanol and 50 mm DTT. The samples were separated on a 10% SDS-PAGE, transferred onto PVDF membranes, and immunoblotted with anti-Panx3.

##### Dephosphorylation Assay

Lysates from HSMM (20 μg) and HEK293T cells transfected with Panx3 (40 μg) were treated with 20 units (for HSMM) or 40 units (for HEK293T+Panx3) of calf intestine phosphatase (CIP) (New England Biolabs) for 1 h at 37 °C. The control lysates (without CIP) were also incubated at 37 °C for 1 h. Samples were boiled and separated on a 10% SDS-PAGE gel and transferred onto PVDF membranes. Membranes were incubated with anti-Panx3 to detect a shift in the banding pattern of its ∼70-kDa immunoreactive species.

##### Differentiation Assay

To induce differentiation, confluent transfected or untransfected HSMM and SkMC were switched to DMEM + 2% horse serum. Cells were fixed for immunolabeling to quantify the percentage of myosin heavy chain (MHC)-positive cells and fusion indices or harvested to quantify the levels of MHC by Western blotting analysis.

After induction of differentiation, cultures were analyzed microscopically to determine the % of MHC-positive cells as well as fusion indices. For this purpose cells were fixed with 3.7% formaldehyde. Permeabilization and blocking of nonspecific binding was done for 1 h with 0.2% bovine serum albumin in PBS containing 0.1% Triton X-100. Cells were incubated with anti-MHC (1:50) with or without anti-Panx1 (1:200) or anti-Panx3 (1:200) or anti-RFP (1:100) for 1 h at room temperature. Cells were washed with PBS containing 0.1% Triton X-100 and were then incubated with secondary antibodies for 45 min at room temperature. Cells were then incubated with Hoechst 33342 for 10 min. After washing, coverslips were applied with Fluoromount G. Fluorescence was viewed with an Olympus IX51 inverted microscope (Olympus). Random fields were analyzed using a 20× objective.

To quantify differentiation and fusion of HSMM, we calculated the differentiation index as the percentage of MHC-positive cells above total nuclei and the fusion index as the percentage of MHC-positive cells containing 2, 3–5, 6–10, or ≥2 nuclei (29). Measurements were obtained from at least three independent experiments. In the experiments in which cells were transfected, differentiation and fusion indexes were determined in transfected cells only. For treatment with probenecid or carbenoxolone, inhibitors were added every 24 h at a final concentration of 1 mm and 100 μm, respectively.

##### Proliferation Assay

Cells were plated in 24-well plates at 25,000 cells per well. For the overexpression experiments, cells were transfected with either GFP as a control, Panx1, or Panx3 on the following day. For the Panx3 knockdown experiments, HSMM were transfected with the control scramble shRNA, Panx3 shRNA63, or Panx3 shRNA64 (Origene Technologies Inc., Rockville, MD). Twenty-four hours post-transfection cells were incubated with 10 μm BrdU (Sigma) for 48 h at 37 °C. Cells were processed for immunohistochemistry as described above, except that DNA was denatured using 2 m HCl for 20 min at room temperature.

In transfection experiments the percentage of BrdU-positive staining nuclei *versus* total nuclei denoted by Hoechst 33342 staining was calculated in transfected cells only. For Panx channel inhibition experiments, probenecid was added every 24 h at a final concentration of 1 mm. The percentage of BrdU-positive staining nuclei *versus* total nuclei was counted in at least three independent experiments.

##### Statistics

All statistical data were analyzed using two-tailed Student's *t* test.

## RESULTS

### 

#### 

##### Panx1 and Panx3 Proteins Are expressed in Skeletal Muscle

**FIGURE 1.**
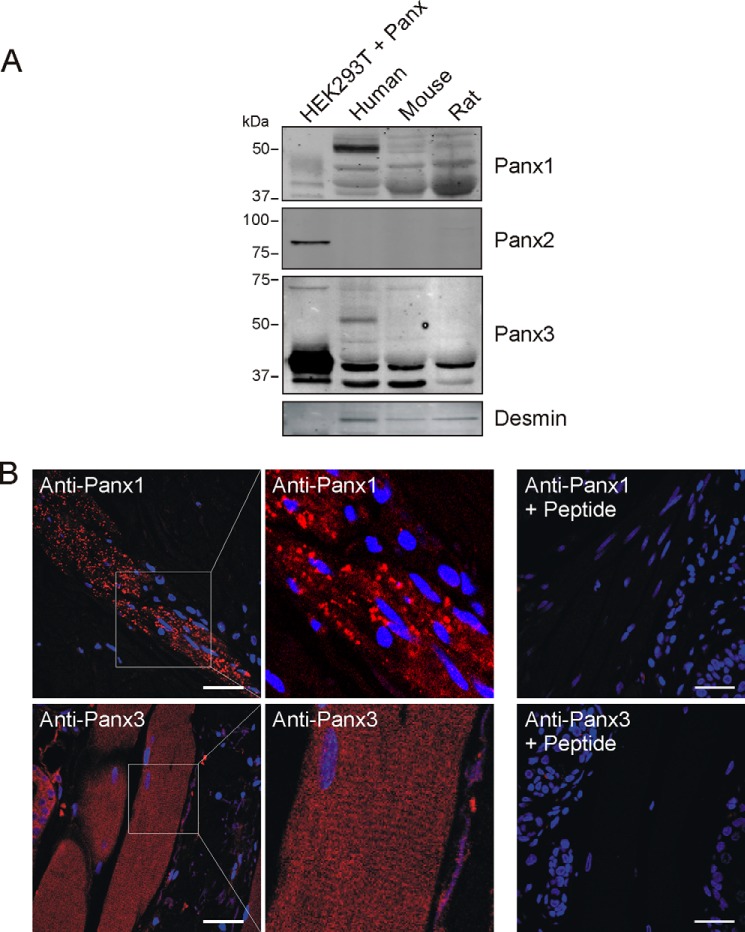
**Panx1 and Panx3 are expressed in the skeletal muscle tissue.** *A*, expression of Panx1, Panx2, and Panx3 proteins in adult human, mouse, and rat skeletal muscle whole tissue lysates was analyzed by Western blotting. Various molecular weight species of Panx1 and Panx3 were expressed, whereas Panx2 was absent or below detectable levels. HEK293T cells transfected with Panx1, Panx2, or Panx3 were used as positive controls. Desmin is a muscle-specific protein. *B*, human skeletal muscle tissue in skin samples was labeled for Panx1 (labeled in *red*) and Panx3 (labeled in *red*). Representative images are shown. Panx1 was detected as a punctate stain, whereas Panx3 was observed as diffuse labeling. Higher magnification micrographs of Panx3 labeling show a striated pattern. Peptide competition revealed loss of specific staining. *Blue* = nuclei; *bars* = 50 μm.

Panx1 transcripts were found strongly expressed in human skeletal muscle (24). At the protein level, Western blot analysis from Riquelme *et al.* (26) showed different molecular weight species of Panx1 in rat muscle, and immunolocalization studies suggested that Panx1 is located at the sarcolemma and at the T-tubules of adult mice and rat fibers (25, 26). In the current study we initially wanted to confirm the presence of Panx1 in this tissue and also determine whether Panx2 and Panx3 are present in the skeletal muscle of human, rat, and mouse by Western blot analysis. As shown in Fig. 1*A*, Panx1 antibodies recognized several species (mainly at ∼38–50 kDa) in human, mouse, and rat skeletal muscle tissue, most likely reflecting various degrees of glycosylation. A similar banding pattern for endogenous Panx1 has been detected in many murine organs (∼41–48 kDa) (3) and as multiple species of ∼43–50 kDa in the rat skeletal muscle (26). As shown in Fig. 1*A*, Panx3 antibodies also recognized several bands in human, mouse, and rat skeletal muscle. All three species express multiple glycosylated forms at ∼43 kDa. Human and mouse skeletal muscle also show the presence of a band at ∼70 kDa. The presence of a Panx3 immunoreactive species at a similar molecular weight (*M*_r_) has also been reported in several murine organs (3), murine skin (6), and organotypic epidermis (7) and in the rat male reproductive tract (8). However, that band was very low or below detectable levels in rat tissue. An additional band at ∼51 kDa was strongly detected by anti-Panx3 in the human skeletal muscle lysate. A similar band has been previously detected in the murine kidney (3). These data differ from that of Riquelme *et al.* (26) reporting that Panx3 is absent in skeletal muscle and may be explained by the use of different Panx3 antibodies. On the other hand, Panx2 was not detected in human, mouse, or rat skeletal muscle lysates (Fig. 1*A*). Skeletal muscles present within human skin sections were then labeled for Panx1 and Panx3. Interestingly, Panx1 was detected as punctate structures, but Panx3 stained diffusely throughout the skeletal muscle fibers and showed striations at higher magnification (Fig. 1*B*). Panx1 and Panx3 labeling were specific as incubation with secondary antibodies alone (data not shown) as well as peptide competition experiments (Fig. 1*B*) abrogated any positive staining. The difference in Panx1 and Panx3 immunolabeling seen here may suggest different functions within the skeletal muscle tissue.

##### Panx1 and Panx3 Levels Are Regulated during Skeletal Muscle Myoblast Differentiation

**FIGURE 2.**
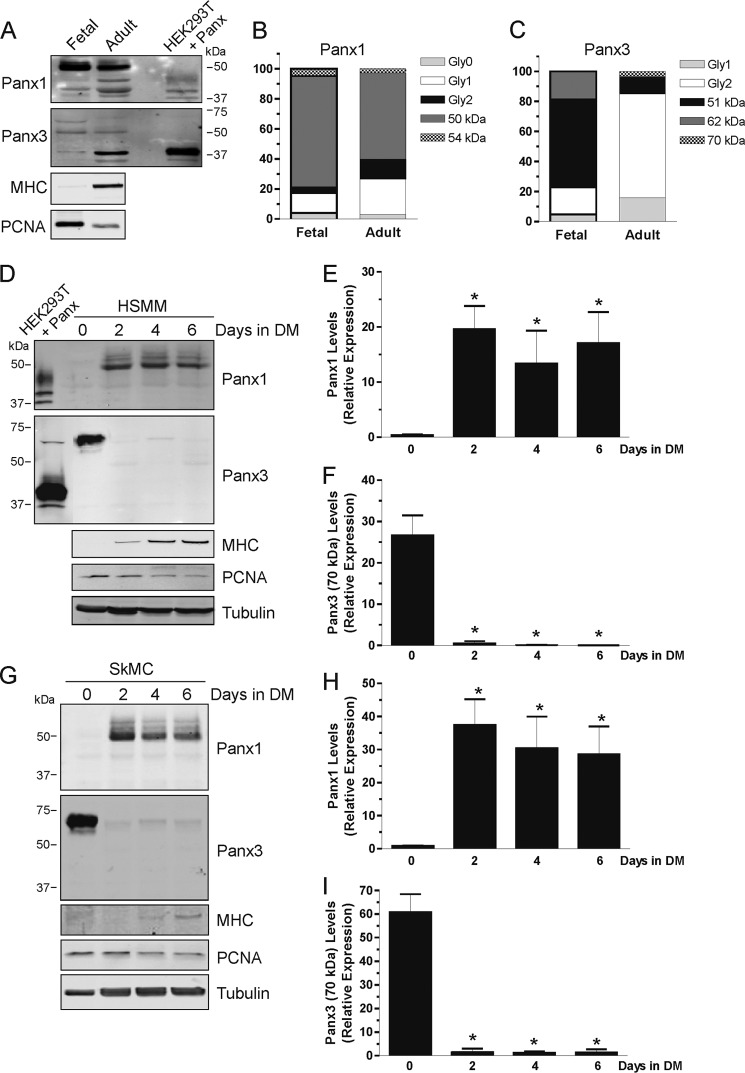
**Panx1 and Panx3 levels are modulated during skeletal muscle myogenic differentiation.** *A*, Panx1 and Panx3 were detected in human fetal and adult skeletal muscle tissue lysates, and their various species were quantified (*B* and *C*). For both Panx1 and Panx3, the higher *M*_r_ species are more abundant in the fetal skeletal muscle, and the banding pattern switches toward lower *M*_r_ forms in the adult tissue. Lysates of HEK293T cells expressing either Panx1 or Panx3 were used as positive controls. MHC and PCNA were detected to show the differentiation and proliferation status of the cells within the tissue, respectively. HSMM (*D*) and SkMC (*G*) were induced to differentiate for 6 days. Panx1 was found at very low levels in undifferentiated cells and was drastically induced during differentiation (*E* and *H*), whereas the ∼70-kDa immunoreactive species of Panx3 was abundant in undifferentiated and proliferative skeletal muscle cells and down-regulated during differentiation (*F* and *I*). As expected, MHC was increased during differentiation, and PCNA levels were reduced. Lysates of HEK293T cells expressing either Panx1 or Panx3 were used as positive controls. Tubulin was used as a loading control.

To start assessing the potential roles of Panx1 and Panx3 in skeletal muscle, we compared Panx expression in human fetal and adult skeletal muscle lysates. During embryonic development, muscle mass increases predominantly by proliferative growth of myoblasts, and genetic programs regulate their differentiation. Postnatally, the contribution of cell proliferation decreases in differentiated myofibers (30). The diminution in cell proliferation in adult compared with fetal skeletal muscle is indicated here by the reduction of proliferating cell nuclear antigen (PCNA) levels, whereas the increase in differentiation is shown by the elevated levels of a marker of terminal differentiation, MHC (Fig. 2*A*). We found that the higher molecular weight species of Panx1 (∼50 kDa) is the main form present in both fetal and adult skeletal muscle. However, it decreases in the adult tissue, whereas the lower molecular forms of Panx1, known as Gly1 and Gly2 (4), become more abundant (Fig. 2, *A* and *B*). These results suggest that the banding pattern of Panx1 shifts from the higher *M*_r_ species in the fetal tissue toward its lower forms in the adult skeletal muscle. Similarly, the main Panx3 species found in the fetal skeletal muscle is at ∼51 kDa. The level of this species decreases in adult tissue, whereas the lower *M*_r_ forms (Gly1 and Gly2) of Panx3 become more abundant (Fig. 2*C*). Interestingly, the levels of the ∼70-kDa immunoreactive species of Panx3 were very low in adults but found in the fetal skeletal muscle at a slightly lower molecular weight (∼62 kDa) (Fig. 2, *A* and *C*). Thus, similar to Panx1, the banding pattern of Panx3 shifts toward lower *M*_r_ species in the adult skeletal muscle tissue. Altogether, these data indicate that the various *M*_r_ species of Panx1 and Panx3 are differentially expressed in fetal compared with adult human skeletal muscle, which may suggest a role in skeletal muscle development possibly linked to proliferation and differentiation processes.

We thus assessed whether Panx levels were modulated during skeletal muscle differentiation. Skeletal myogenesis is a highly ordered process that includes cell commitment, irreversible cell cycle withdrawal, phenotypic differentiation that is indicated by the induction of MHC, and cell fusion (31). Skeletal myogenesis can be induced in culture by depriving cycling myoblasts of serum, which then form multinucleated myotubes that are irreversibly postmitotic (31). Human primary skeletal muscle myoblasts (HSMM) and skeletal muscle cells (SkMC) were thus induced to differentiate by serum deprivation, and the levels of Panx1 and Panx3 were monitored during this process. As expected, the levels of MHC were very low at day 0 but increased during differentiation, whereas PCNA levels were high in undifferentiated cells and down-regulated during the differentiation process (Fig. 2, *D* and *G*). Interestingly, we found that the levels of Panx1 were very low in undifferentiated HSMM (Fig. 2, *D* and *E*) and SkMC (Fig. 2, *G* and *H*) and augmented during differentiation. By contrast, the levels of the ∼70-kDa immunoreactive species of Panx3 were high in proliferating undifferentiated HSMM and SkMC and drastically down-regulated during their differentiation (Fig. 2, *D*, *F*, *G*, and *I*). The Gly1 and Gly2 (∼43 kDa) species of Panx3, which were the most prominent in the adult skeletal muscle tissue, were either very low or below detectable levels in HSMM and SkMC during the 6-day time course of the differentiation assay. This might suggest that these low molecular weight forms of Panx3 are expressed further along the differentiation process, and although myoblasts differentiate and express markers such as MHC and can form multinucleated myotubes to a certain degree *in vitro*, formation of contractile myotubes was not observed. Altogether, these results indicate a modulation of Panx1 and Panx3 levels during myogenic differentiation, suggesting that these proteins may play a role in the regulation of skeletal muscle myoblast differentiation and/or proliferation.

##### Treatment with N-Glycosidase F and Sialidase A Alters the Electrophoretic Motility of the ∼70-kDa Immunoreactive Species of Panx3

**FIGURE 3.**
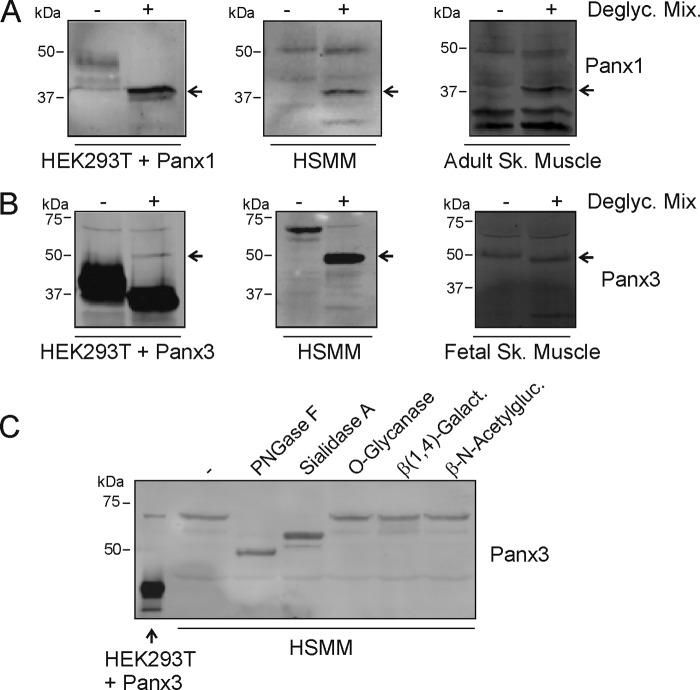
**Treatment with *N*-glycosidase F and sialidase A alter the electrophoretic motility of the ∼70-kDa immunoreactive species of Panx3.** *A*, deglycosylation using a mix of enzymes (*N*-glycosidase F, *O*-glycanase, neuraminidase (sialidase), β(1,4)-galactosidase, and β-*N*-acetylglucosaminidase) caused a shift in electrophoretic mobility (*arrow*) of the higher *M*_r_ form of Panx1 (∼50 kDa) expressed in differentiated HSMM and adult skeletal muscle tissue, similar to Panx1 transfected in HEK293T cells. *B*, incubation with that same mix of deglycosylation enzymes also caused a shift in the electrophoretic motility (*arrow*) of the ∼70-kDa immunoreactive species of Panx3 expressed in undifferentiated HSMM and human fetal skeletal muscle tissue and to the lower *M*_r_ form of Panx3 transfected in HEK293T cells. *C*, treatment with *N*-glycosidase F (*PNGase F*) and sialidase A independently resulted in a shift in the electrophoretic mobility of the ∼70-kDa immunoreactive species of Panx3 in HSMM but not with *O*-glycanase, β(1–4)-galactosidase, or β-*N*-acetylglucosaminidase.

As the lower molecular weight forms of Panxs were faint or below detectable levels in human primary skeletal muscle myoblasts in culture, we wanted to confirm that the higher molecular weight bands detected correspond to Panx1 and Panx3. To this end, HSMM and adult skeletal muscle tissue lysates were treated with a mixture of deglycosylation enzymes (*N*-glycosidase F, *O*-glycanase, neuraminidase (Sialidase), β(1,4)-galactosidase, and β-*N*-acetylglucosaminidase) and submitted to Western blotting. After deglycosylation, the endogenous ∼50-kDa protein species detected by anti-Panx1 in HSMM migrated further at ∼38 kDa (Fig. 3*A*). As a positive control, we show that the more classical Panx1 species also migrated further after treatment of Panx1-expressing HEK293T cell lysate with deglycosylation enzymes (Fig. 3*A*) (4). As for Panx3, the ∼70-kDa immunoreactive species detected by anti-Panx3 in HSMM and human fetal skeletal muscle lysates also migrated further into a predominant band at ∼50–51 kDa after treatment with deglycosylation enzymes (Fig. 3*B*). As a positive control, we show that this band was also present after submitting HEK293T over-expressing Panx3 to deglycosylation in addition to bands ranging from ∼35–40 kDa (Fig. 3*B*) (7). Because these results suggest that the ∼70-kDa immunoreactive species of Panx3 may have post-translational modifications that are different from the classical ∼43 kDa form, we wanted to better understand and characterize the glycosylation status of this higher *M*_r_ species. To this end, HSMM lysates were treated with the same enzymes but individually. As shown in Fig. 3*C*, the ∼70-kDa species of Panx3 migrated further after treatment with *N*-glycosidase F and sialidase A but not with *O*-glycanase, β(1,4)-galactosidase, or β-*N*-acetylglucosaminidase. However, *O*-glycosylation cannot be completely ruled out as the presence of sialic acids can block the action of *O*-glycanase. These results thus indicate that the ∼70-kDa immunoreactive species of Panx3 is modified by *N*-glycosylation and sialylation. Taken together these data suggest that the higher molecular weight species detected in HSMM and human skeletal muscle correspond to highly glycosylated species of endogenous human Panx1 and Panx3.

##### The Electrophoretic Mobility of the ∼70-kDa Species of Panx3 Is Altered by Alkaline Phosphatase Treatment

**FIGURE 4.**
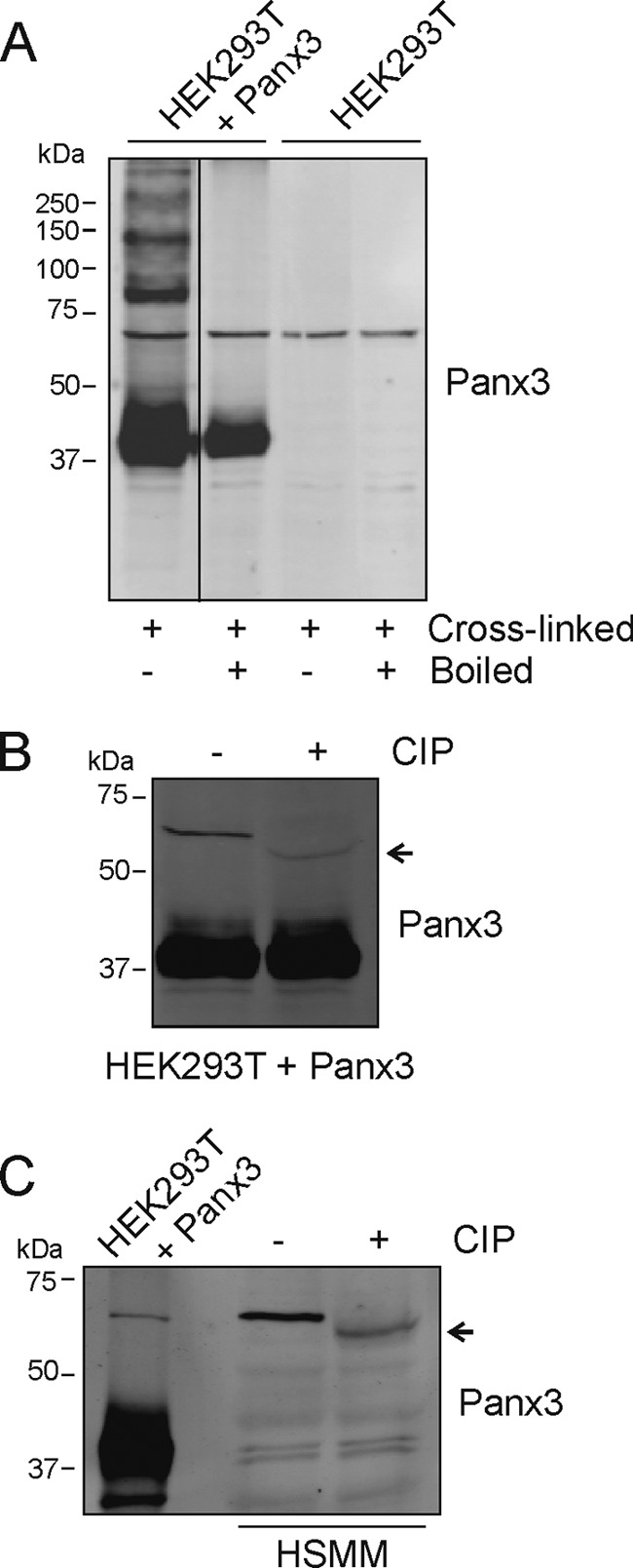
**Treatment with alkaline phosphatase alters the electrophoretic motility of the ∼70-kDa immunoreactive species of Panx3.** *A*, parental HEK293T cells and HEK293T transfected with Panx3 were solubilized with Triton X-100, cross-linked with DSP, separated by SDS-PAGE, and immunoblotted with anti-Panx3. After the cross-links were reversed by boiling the samples in the presence of DTT, only the ∼43-kDa (monomer) and ∼70-kDa immunoreactive species of Panx3 were still present. Lysates of HEK293T cells transfected with Panx3 (*B*) or HSMM (*C*) were treated with CIP and submitted to SDS-PAGE. Although the electrophoretic motility of the lower *M*_r_ form of Panx3 was not affected (*B*), its ∼70-kDa immunoreactive species migrated further (*arrow*) after treatment of HEK293T and HSMM lysates with CIP. Representative Western blots of three independent experiments are shown.

Because of its apparent *M*_r_, the ∼70-kDa immunoreactive species of Panx3 could possibly correspond to a Panx3 dimer. However, heating in combination with denaturing agents such as SDS and β-mercaptoethanol with or without DTT could not reduce it to a lower *M*_r_ form (data not shown). To further explore the possibility of a Panx3 dimer, we used the homobifunctional, amine-reactive reagent DSP to cross-link Panx3 following a method that has been used to show the oligomeric state of Panx1 (5). Triton X-100-solubilized extracts of wild-type or HEK293T cells transfected with Panx3 (∼43 kDa) were treated with the amino-reactive reagent DSP. After treatment with DSP, multiple bands were observed including the Panx3 monomer, the ∼70-kDa immunoreactive species, and bands at ∼85 kDa and higher (Fig. 4*A*). When the samples were boiled in the presence of DTT to reverse the cross-links, only the Panx3 monomer (∼43 kDa) and its ∼70-kDa immunoreactive species were still observed, suggesting that this band does not correspond to a Panx3 dimer. However, one of the bands detected after incubation with DSP at around 85 kDa may correspond to a dimer based on its size and its disappearance after boiling.

As the ∼70-kDa immunoreactive species of Panx3 does not appear to correspond to a Panx3 dimer, it may have other post-translational modifications that differ from the classical lower *M*_r_ species in addition to its sialylation. Although Panx1 and Panx3 have predicted phosphorylation sites (3), the banding pattern of Panx1 overexpressed in NRK cells was not altered after treatment with alkaline phosphatase (3). However, Panx1 was recently suggested to be phosphorylated on serine and threonine residues during potentiation of skeletal muscle contraction (26). Because the molecular mass of the ∼70-kDa immunoreactive species of Panx3 seems to be slightly different in the fetal *versus* adult skeletal muscle tissue and a faint band of a lower molecular weight could also be detected in HSMM and SkMC lysates, we tested whether that shift could be due to phosphorylation. To this end, lysates of HEK293T cells transfected with Panx3 were treated with CIP and submitted to SDS-PAGE. Although the ∼43-kDa species of Panx3 were not altered by this treatment, the ∼70-kDa immunoreactive species of Panx3 migrated further after incubation with CIP (Fig. 4*B*). Similar results were also obtained using HSMM lysates (Fig. 4*C*). Although the exact identity of the ∼70-kDa immunoreactive species of Panx3 remains unknown, our data suggest that it does not correspond to a Panx3 dimer but instead to a species that is *N*-glycosylated, sialylated, and phosphorylated.

##### Panx1 Overexpression Promotes Skeletal Muscle Myoblast Differentiation

**FIGURE 5.**
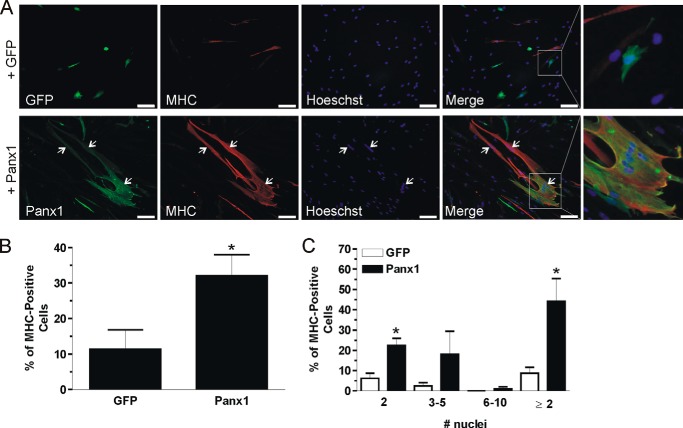
**Overexpression of Panx1 accelerates human primary skeletal muscle myoblast differentiation.** HSMM were transfected with Panx1 (labeled in *green*) or GFP and placed in differentiation medium and examined 2 days later for MHC (labeled in *red*) expression and myotube formation. Representative pictures of three independent experiments are shown in *A*. When compared with cells expressing GFP, a higher proportion of Panx1-transfected cells were MHC-positive (*B*), and among those cells, a higher percentage contained two or more nuclei indicative of fusion (*A*, *arrow*), which was quantified in *C. Blue* = nuclei; *bars* = 50 μm. *, *p* < 0.05 compared with GFP.

Because Panx1 levels were increased during skeletal muscle differentiation, it is tempting to speculate that its expression promotes this process. To verify this hypothesis, Panx1 was overexpressed in HSMM and tested for its effect on their differentiation. HSMM were chosen for this assay as their ability to differentiate was found more consistent than that of SkMC. HSMM were thus transfected with either GFP or Panx1 and induced to differentiate for 2 days. Transfected cells were then examined for their expression of MHC and the formation of multinucleated myotubes as markers for terminal differentiation (Fig. 5*A*). After 2 days in differentiation media, a significant higher proportion of Panx1-transfected HSMM were MHC-positive (Fig. 5*B*) and contained two or more nuclei (Fig. 5*C*) when compared with control cells transfected with GFP, which did not show evidence of fusion yet. These results thus indicate that Panx1 promotes earlier skeletal muscle differentiation.

##### Probenecid and Carbenoxolone Inhibit Skeletal Muscle Myoblast Differentiation

**FIGURE 6.**
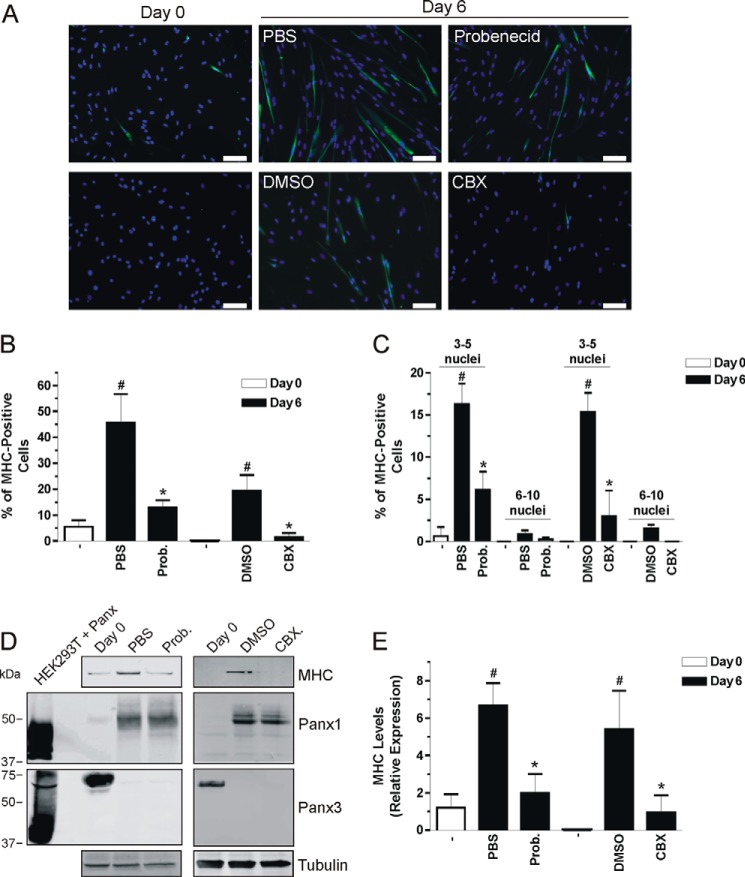
**Probenecid and carbenoxolone inhibit human primary skeletal muscle myoblast differentiation.** HSMM were placed in differentiation medium for 6 days in the presence or absence of 1 mm probenecid (or its vehicle control, PBS) or 100 μm CBX (or its vehicle control, DMSO), and examined for MHC (labeled in *green*) expression and myotube formation. Representative pictures of three independent experiments are shown in *A*. Probenecid and CBX significantly reduced the percentage of MHC-positive cells after 6 days of differentiation (*B*) as well as the number of nuclei in MHC-positive cells (*C*). After 6 days in differentiation medium, HSMM were also analyzed for MHC levels. The increase in MHC seen after 6 days in differentiation medium was significantly reduced when cells were incubated with probenecid or CBX (*D* and *E*) without affecting the levels of endogenous Panx1 and Panx3 (*D*). Tubulin was used as a loading control. *Blue* = nuclei; *bar* = 100 μm; *, *p* < 0.05 compared with *Day 0*.

To confirm the role of Panx1 channels in differentiation, HSMM were treated with probenecid and CBX (32, 33) and tested for their effect on this process. As shown in Fig. 6, *A* and *B*, <10% of HSMM were positive for MHC at day 0, which increased to 20–45% after 6 days in differentiation media (DM). Among those MHC-positive cells, about 15% contained 3–5 nuclei after 6 days in DM, which is indicative of fusion, compared with <1% at day 0 (Fig. 6*C*). However, when cells were treated with 1 mm probenecid or 100 μm CBX during the 6-day incubation in DM, the percentage of cells that were positive for MHC was drastically reduced (Fig. 6*B*) as well as the percentage of MHC-positive cells that had 3–5 nuclei (Fig. 6*C*). Taken together, these results suggest that inhibition of Panx1 channels impairs skeletal muscle myoblast differentiation.

These results were next confirmed by Western blotting as the levels of MHC, which were significantly increased in HSMM after 6 days in DM when compared with day 0, were drastically reduced when cells were incubated with probenecid or CBX (Fig. 6, *D* and *E*). This effect was not due to an alteration of the modulation of endogenous Panx1 and Panx3 levels that occur during differentiation (Fig. 6*D*). These results are thus in accordance with the Panx1 overexpression data and altogether indicate that Panx1 channels regulate skeletal muscle differentiation.

##### Panx1 Does Not Regulate Skeletal Muscle Myoblast Proliferation

**FIGURE 7.**
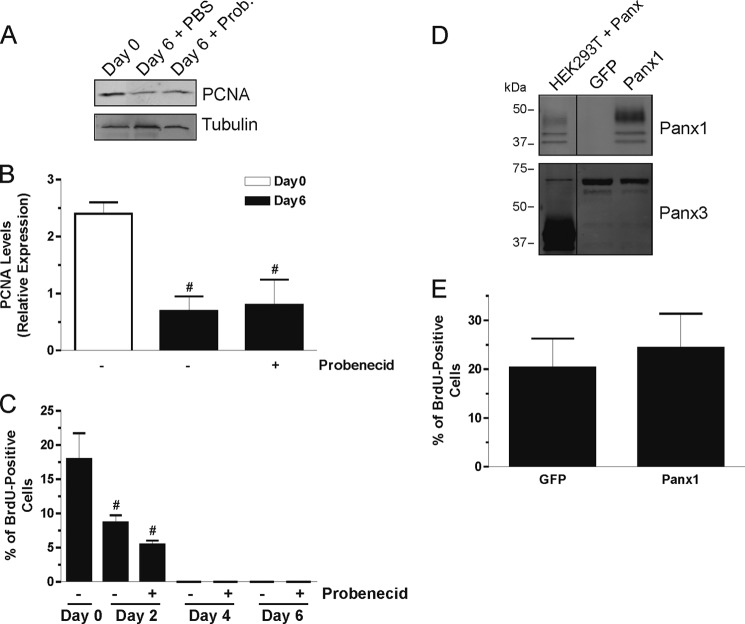
**Panx1 does not regulate skeletal muscle myoblast proliferation.** HSMM were placed in differentiation medium for 6 days in the presence or absence of probenecid (*Prob*) and examined for PCNA levels by Western blotting (*A*). The decrease of PCNA seen after cells are placed in DM for 6 days was not affected by probenecid (*A* and *B*). BrdU incorporation assays showed a reduction of BrdU-positive cells after 2 days in DM, which was not affected by probenecid (*C*). After 2 days no BrdU-positive cells could be detected either in the presence or absence of the inhibitor. HSMM were transfected with Panx1 or GFP as a control, which did not affect the expression of endogenous Panx3 (*D*). Cells were then submitted to a BrdU assay (*E*), which revealed that Panx1 overexpression did not affect BrdU incorporation in growth medium. Tubulin was used as a loading control. #, *p* < 0.001 compared with *Day 0*.

As cell differentiation and proliferation are generally exclusive processes, we next wanted to determine whether the inhibitory effect of Panx channel blockers on differentiation involves regulation of cell proliferation. To this end the levels of PCNA were assessed during HSMM differentiation in the absence or presence of the inhibitors. The reduction of PCNA levels that occurs during differentiation was not affected by probenecid (Fig. 7, *A* and *B*) or CBX (data not shown). To confirm these results, a BrdU incorporation assay was performed during HSMM differentiation with or without probenecid. After 2 days in DM, the percentage of BrdU-positive cells was significantly reduced when compared with day 0 but was not affected by the inhibitor (Fig. 7*C*). After that end point, no BrdU-positive cells could be detected in any condition. These data suggest that these inhibitors did not alter differentiation of skeletal muscle myoblasts by preventing them from exiting the cell cycle.

We also assessed whether Panx1 plays a role in serum-induced cell proliferation by overexpressing Panx1 in HSMM, which did not affect the levels of endogenous Panx3 (Fig. 7*D*), and submitting the cells to a BrdU incorporation assay in normal growth media. As shown in Fig. 7*E*, Panx1 overexpression did not affect BrdU incorporation when compared with control cells. These results thus indicate that although Panx1 regulates the differentiation of skeletal muscle myoblasts, it is not involved in the regulation of their proliferation.

##### Overexpression of the ∼43-kDa Panx3 Species Inhibits Proliferation and Promotes Differentiation of Skeletal Muscle Myoblasts

**FIGURE 8.**
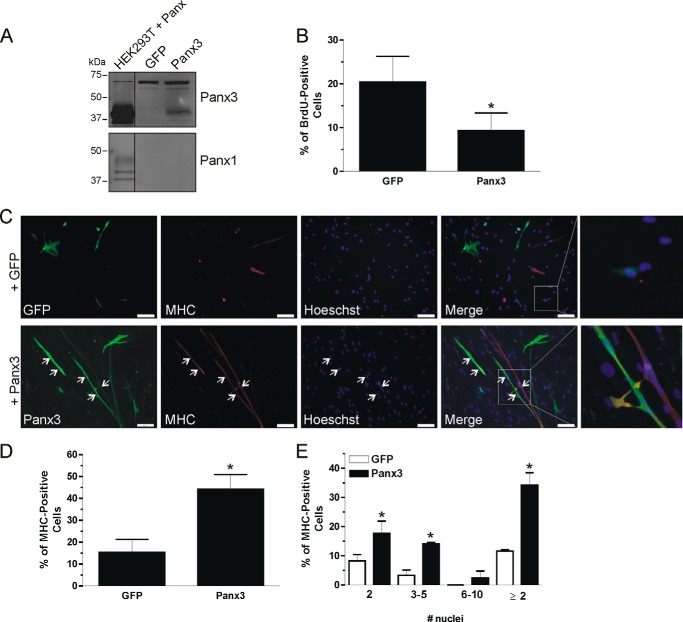
**Overexpression of Panx3 (∼43-kDa species) reduces human primary skeletal muscle myoblast proliferation and promotes their differentiation.** Transfection of Panx3 in HSMM resulted in the overexpression of the ∼43-kDa species without affecting the levels of the ∼70-kDa immunoreactive form of Panx3 (*A*). Transfection of Panx3 did not increase the expression of endogenous Panx1, which was very low when cells were in growth medium (*A*). These cells were submitted to a BrdU incorporation assay, which revealed that the overexpression of the lower *M*_r_ species of Panx3 resulted in a significant reduction of cell proliferation (*B*). After being placed in differentiation medium for 2 days, these cells were examined for expression of MHC (labeled in *red*) and formation of myotubes (*C*). A higher percentage of cells transfected with Panx3 (labeled in *green*) were MHC-positive when compared with GFP (*D*). Among those MHC-positive cells, a higher proportion contained at least two or more nuclei when overexpressing Panx3 (*C*, *arrow*), which was quantified in *E. Blue* = nuclei; *bar* = 100 μm; *, *p* < 0.05 compared with GFP.

We next overexpressed Panx3 in HSMM to assess its role in skeletal muscle myoblast proliferation and differentiation. As shown in Fig. 8*A*, transfection of Panx3 led to the overexpression of the lower molecular weight form of the protein, which was predominantly expressed in the adult skeletal muscle tissue. On the other hand, the levels of the endogenous ∼70-kDa immunoreactive species of Panx3 remained unchanged. The expression levels of endogenous Panx1 were also not altered by transfection of Panx3 (Fig. 8*A*). To determine the effect of Panx3 (∼43 kDa) overexpression on HSMM proliferation, a BrdU incorporation assay was performed in growth media. As shown in Fig. 8*B*, we found that the percentage of BrdU-positive cells was significantly lower in Panx3-transfected cells when compared with control.

We next wanted to investigate the role of Panx3 in skeletal muscle differentiation by overexpressing the ∼43-kDa species of Panx3 in HSMM. After being placed in DM for 2 days, a higher proportion of Panx3-transfected HSMM was found to be MHC-positive (Fig. 8, *C* and *D*) and containing 2–5 nuclei (Fig. 8, *C* and *E*) when compared with control cells transfected with GFP, indicating that Panx3 promotes skeletal muscle differentiation. Altogether, these results thus suggest that the lower molecular weight form of Panx3 inhibits skeletal muscle myoblast proliferation and promotes their differentiation.

##### Knockdown of the ∼70-kDa Immunoreactive Species of Panx3 Inhibits Skeletal Muscle Myoblast Proliferation without Inducing Their Differentiation

**FIGURE 9.**
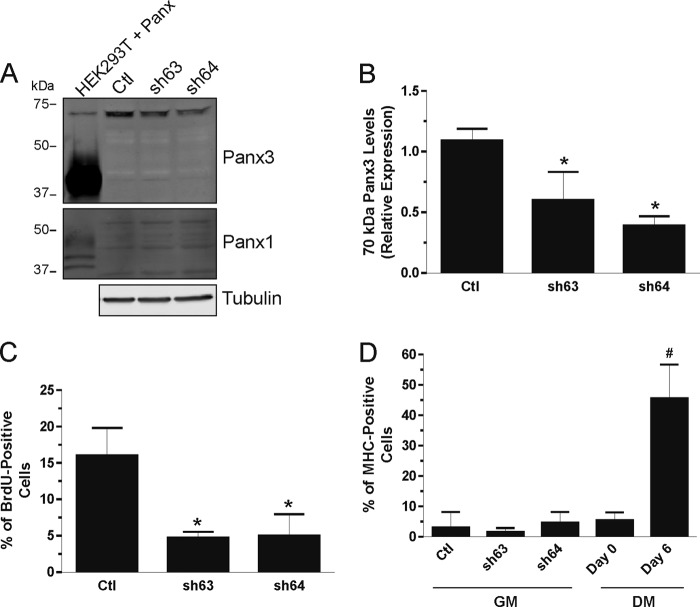
**Knockdown of the ∼70-kDa immunoreactive species of Panx3 inhibits skeletal muscle myoblast proliferation without inducing their differentiation.** Transfection of two Panx3 shRNAs (sh63 and sh64) resulted in a significant reduction of the ∼70-kDa immunoreactive species of Panx3 compared with the control scramble shRNA (*Ctl*) (*A* and *B*) without modifying the levels of Panx1 nor the ∼43-kDa species of Panx3 (*A*). BrdU incorporation assay showed that knockdown of the ∼70-kDa species reduces HSMM proliferation in growth medium (*GM*) (*C*) without triggering their differentiation (*D*). Undifferentiated (*Day 0*) and differentiated (*Day 6* in DM) HSMM were used as comparison. Tubulin was used as a loading control. *, *p* < 0.05 compared with the Ctl shRNA; #, *p* < 0.001 compared with *Day 0*.

The ∼70-kDa Panx3 immunoreactive band detected in undifferentiated HSMM and skeletal muscle tissue has also been detected in murine organs (3), murine skin (6), rat organotypic epidermis (6, 7), and rat male reproductive tract (8). In organotypic epidermis, this band was recognized by three different anti-Panx3 antibodies and also corresponds to a glycoprotein (7). We now demonstrate that the endogenous levels of the ∼70-kDa immunoreactive species of Panx3 can be significantly knocked down using two different shRNAs against Panx3 (Fig. 9, *A* and *B*), which provides further evidence that it corresponds to a Panx3 species. Panx1 levels and Panx3 lower *M*_r_ species remained very low or below detectable levels in cells transfected with these Panx3 shRNAs (Fig. 9*A*).

Interestingly, our results indicate that proliferative undifferentiated HSMM and SkMC express the ∼70-kDa immunoreactive species of Panx3 that was drastically down-regulated during differentiation (Fig. 2, *D*, *F*, *G*, and *I*). These data suggest that this species may play a role in keeping skeletal muscle myoblasts in a proliferative state. To test this hypothesis, HSMM were transfected with Panx3 shRNAs and submitted to a proliferation assay in growth medium, a condition in which the cells mainly express the ∼70-kDa immunoreactive species of Panx3. As shown in Fig. 9*C*, transfection with Panx3 shRNAs significantly reduced the proportion of BrdU-positive cells when compared with HSMM transfected with scramble shRNA (*Ctl*). However, this reduction of cell proliferation was not sufficient by itself to trigger differentiation, as shown by the low percentage of MHC-positive cells, which was comparable with that of undifferentiated cells (*Day 0* in DM) (Fig. 9*D*). These results thus suggest that the ∼70-kDa immunoreactive species of Panx3 is involved in keeping undifferentiated skeletal muscle myoblasts in a proliferative state.

## DISCUSSION

We report here that various molecular weight species of both Panx1 and Panx3 are expressed in human and rodent skeletal muscles as well as in primary human skeletal muscle cells. Skeletal muscle abundantly expressed a higher molecular mass form (∼50 kDa) of Panx1, which is likely due to a higher degree of glycosylation, as well as its expected lower molecular weight species (Gly0, Gly1, and Gly2). That ∼50-kDa species was also the main form of Panx1 detected in human primary skeletal muscle cells and myoblasts, suggesting that the lower molecular weight species of Panx1 are expressed further along the differentiation process, which would also be consistent with the expression pattern seen in fetal *versus* adult skeletal muscle tissue. A similar trend was observed for Panx3 as its lower molecular weight species were more abundant in the adult than in the fetal skeletal muscle. Indeed, we found that the adult skeletal muscle tissue mainly expresses its expected low molecular weight species (Gly1 and Gly2) and to a lower extent immunoreactive species at ∼51 and ∼70 kDa. The main species observed in the fetal tissue homogenate were at ∼51 and ∼62 kDa. The ∼51-kDa form may correspond to an intermediate glycosylated species as deglycosylation of the ∼70-kDa form generated a band at a similar size (Fig. 3, *B* and *C*), whereas the band with an apparent *M*_r_ of ∼62 kDa may represent a non-phosphorylated or dephosphorylated form of the ∼70-kDa species. Although the exact identity of the ∼70-kDa Panx3 immunoreactive species is currently under investigation, it possesses the intrinsic characteristics of Panxs as it is glycosylated, it is recognized by three different antibodies directed against Panx3 (7), and our new data now show that its levels can be reduced using Panx3 shRNAs. Altogether, these findings strongly suggest that this band corresponds to Panx3. Furthermore, our data suggest that it is not a Panx3 dimer but instead a form of Panx3 that is likely modified differently than the classical low *M*_r_ form (∼43 kDa) of Panx3 as our findings suggest that in addition to glycosylation, it is also modified by sialylation and phosphorylation.

**FIGURE 10.**
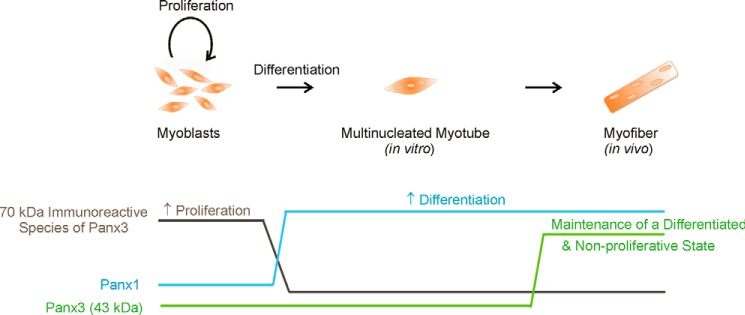
**Panx1 and Panx3 levels are modulated during skeletal muscle myoblast differentiation and regulate this process as well as myoblast proliferation.** Proliferative undifferentiated skeletal muscle myoblasts express high levels of the ∼70-kDa immunoreactive species of Panx3, whereas its lower *M*_r_ species or Panx1 were very low. Its levels were drastically diminished during differentiation, suggesting that the ∼70-kDa immunoreactive species of Panx3 may play a role in keeping undifferentiated skeletal muscle myoblasts in a proliferative state. On the other hand, the levels of the low *M*_r_ form of Panx3 are very low in skeletal muscle myoblasts in culture, slightly detected in fetal skeletal muscle tissue, and further increased in the adult. Its expression promotes human skeletal muscle myoblast differentiation and inhibits their proliferation. Altogether, our data suggest that the lower *M*_r_ species of Panx3 play a role in maintaining differentiated myoblasts in a differentiated and non-proliferative state. As for Panx1, its protein levels were drastically augmented during skeletal muscle myoblast differentiation and promote this process independently of cell proliferation.

In addition to their differential expression in fetal *versus* adult skeletal muscle tissue, we also show here that Panx levels are regulated during skeletal muscle myoblast differentiation *in vitro*. During development, embryonic muscle mass increases predominantly by proliferative growth of myoblasts that differentiate to eventually generate fully differentiated myofibers in adults. Skeletal muscle terminal differentiation is a temporally ordered process that follows an organized sequence of events including commitment, cell cycle withdrawal, expression of muscle-specific proteins, and myoblast fusion to form multinucleated myotubes (34). Our results clearly demonstrated that both Panx1 and Panx3 regulate skeletal muscle myoblast differentiation and proliferation, which are summarized in our proposed model in Fig. 10. Indeed, we have found that the levels of Panx1 are very low in undifferentiated and proliferative skeletal muscle myoblasts but increased drastically during their differentiation. Accordingly, its overexpression accelerated skeletal muscle myoblast differentiation through a process that likely involves its channel functions but did not regulate myoblast proliferation. As for Panx3, its lower molecular weight form was not detected or was below detectable levels in both undifferentiated and differentiated HSMM but was expressed in human skeletal muscle tissue, which may suggest that it is expressed further along the differentiation process. This form was also more abundant in adult *versus* fetal skeletal muscle tissue. When overexpressed in HSMM, it promoted their differentiation and inhibited their proliferation, suggesting that it may play a role in maintaining the skeletal muscle myoblasts in a differentiated and non-proliferative state. On the other hand, the ∼70-kDa immunoreactive species of Panx3 was found highly expressed in undifferentiated HSMM. Its levels were drastically down-regulated during differentiation, and its knockdown significantly inhibited HSMM proliferation, which may thus suggest a role in keeping the undifferentiated skeletal muscle myoblasts in a proliferative state. Altogether, our results thus indicate that various species of Panx1 and Panx3 are expressed in skeletal muscle tissue and that they differentially regulate skeletal muscle myoblast differentiation and proliferation.

Although we are currently investigating the mechanisms by which Panx1 and Panx3 regulate skeletal muscle myoblast differentiation, the inhibition of HSMM differentiation by probenecid and CBX suggests that Panx1 channels are involved in this process. Although probenecid reduced the Panx3-mediated dye uptake in transfected HEK293T cells (data not shown), the effect of this inhibitor on HSMM differentiation is most likely not due to Panx3 channels as both the ∼43- and ∼70-kDa species are very low or below detectable levels in these conditions (Fig. 6*D*). Before the discovery of Panxs, it was shown that treatment of L6 rat myoblasts with β-glycyrrhetinic acid, which is a blocker of connexin and Panx channels, inhibited the induction of myogenin and MRF4 expression that occurs during differentiation (35). Interestingly, among the molecular players involved in skeletal muscle differentiation, it has been reported that extracellular ATP, activation of purinoreceptors (P2X and P2Y), and increase in intracellular Ca^2+^ concentration are also required (34, 36, 37). Araya *et al.* (34) hypothesized in 2004 that extracellular ATP activates P2X receptors on the cell surface and that the Ca^2+^ influx generated through the activated P2X receptor activates the phospholipase δ that yields inositol triphosphate. At that time it was suggested that the inositol triphosphate diffuses to neighboring cells through gap junctions where it induces release of Ca^2+^ from intracellular stores and that increase in free intracellular Ca^2+^ levels leads to myogenin expression and induction of C_2_C_12_ differentiation (34). Since then Panx1 has been shown to be involved in the ATP release of many cell types (for review see Ref. 1) including skeletal myotubes (27). Interestingly, ATP release was recently shown not to occur in muscles from Panx1 knock-out mice (26). As Panx1 is now known for its involvement in the initiation and propagation of Ca^2+^ wave signaling through P2X and P2Y receptors (for review see Ref. 1), it is tempting to speculate that Panx1 may play a role in the extracellular ATP release and Ca^2+^ wave propagation required for skeletal myogenic differentiation.

Although Panx1 channel functions have been studied in many cell systems, much less is known about Panx3. It has been previously shown to regulate chondrocyte differentiation and proliferation, possibly through regulation of intracellular ATP and cAMP levels (21). It has also been reported to promote osteoblast differentiation by acting as an ER Ca^2+^ channel and hemichannel that releases ATP into extracellular space, activating purinergic receptors and the subsequent PI3K-Akt signaling (23). Although controversial, Panx3 was also suggested to act as gap junction channel in that study. Very recently, Panx3 was shown to regulate proliferation of osteoprogenitor cells by regulating Wnt and p21 signaling (22). As for its ∼70-kDa immunoreactive species, only data regarding its expression profile had been reported to date (3, 6–8), and its identity and thus functions were still unknown. Because a similarly sized band has been observed in fetal bovine serum (FBS), FBS may represent a source of error when interpreting results from cell culture (38). However, the reduction of the ∼70-kDa immunoreactive species of Panx3 observed when changing HSMM and SkMC from growth medium, which contains FBS, to differentiation medium, which instead contains horse serum, was not seen with other cell types (data not shown). This suggests that the regulation of this species observed here is linked to processes involved in skeletal muscle differentiation. It would thus be very useful to verify the presence or absence of this ∼70-kDa species in Panx3 knock out mice, determining its sequence, and possibly performing overexpression experiments to further understand its possible functions in skeletal muscle myoblast proliferation, differentiation, and development.

In addition to the modulation of Panx species levels during skeletal muscle myoblast differentiation, our results also suggest a regulation of their glycosylation and phosphorylation status during this process. The glycosylation of Panxs has been reported as being important for their proper trafficking and cellular localization as well as for their intermixing, which can potentially regulate their channel function (3–5). In addition to the interaction between Panx family members, other proteins have been shown to interact with Panxs such as the dihydropyridine receptor that co-precipitated with Panx1 and the P2Y_2_ receptor using skeletal myotube lysates (27). Interestingly, Panx phosphorylation has only been reported in skeletal muscle so far. Immunoprecipitation studies using whole rat skeletal muscle lysates suggest that Panx1 is phosphorylated on serine and threonine residues during potentiation of contraction (26). Our results indicate that in addition to being glycosylated and phosphorylated, the ∼70-kDa immunoreactive species of Panx3 is also sialylated. Although the role of this modification is not known in the context of Panxs, sialic acid residues can modulate voltage-dependent channel gating directly (for review see Ref. 39). The potential roles of Panx post-translational modifications and protein interactions in skeletal muscle myoblast differentiation and proliferation are still unknown, but they may be involved in the regulation of Panx channel function during these processes.

As more than one Panx species is expressed concurrently during skeletal muscle myoblast differentiation and likely during skeletal muscle development, the differential modulation of their levels, post-translational modifications, and functions may govern the conversion of these cells from an undifferentiated proliferative state to a differentiated one thereby regulating skeletal muscle cell fate. Collectively our results suggest that a fine balance between Panx species may exist during skeletal muscle developmental mechanisms, such as myoblast differentiation and proliferation. Because skeletal muscle myoblast proliferation and differentiation are crucial events involved in muscle development and regeneration, Panxs may play a important role in these fundamental physiological processes as well as in conditions such as muscle wasting and cachexia.

## Footnotes

2 The abbreviations used are:

Panx: pannexin
CBX: carbenoxolone
HSMM: human skeletal muscle myoblast(s)
SkMC: skeletal muscle cell(s)
DSP: dithiobis(succinimidylpropionate)
CIP: calf intestinal (alkaline) phosphatase
PCNA: proliferating cell nuclear antigen
DM: differentiation media.
